# 138. Implementation of an Antimicrobial Stewardship Order Set in the Emergency Department at a Quaternary Care Facility

**DOI:** 10.1093/ofid/ofab466.340

**Published:** 2021-12-04

**Authors:** Salma M . Al Shaqfa, Rania M El Lababidi, Wasim S El Nekidy, Mohamed Hisham, Rama Nasef, Hazem Elrefaei, Fernanda Bonilla

**Affiliations:** 1 Cleveland Clinic Abu Dhabi, Abu Dhabi, Abu Dhabi, United Arab Emirates; 2 Cleveland Clinic Abu Dhabi and Cleveland Clinic Lerner College of Medicine, Abu Dhabi, Abu Dhabi, United Arab Emirates

## Abstract

**Background:**

Implementation of antimicrobial stewardship (AS) interventions in the emergency department (ED) has been associated with improved patient outcomes. One potentially promising AS strategy is the implementation of an ED-specific, evidence-based antimicrobial order set. In this study, we aimed to examine the impact of implementing an ED-specific order set (EDOS) on the appropriateness of empiric antimicrobial therapy.

**Methods:**

We conducted a pre-post quasi experimental study on 160 adult patients presenting to the ED with suspected or confirmed common infections at our quaternary healthcare facility. The EDOS was implemented in December 2020, providing evidence-based recommendations for the management of common infectious diseases. Data was collected between September 2019 and March 2020 for the pre-EDOS implementation group and between January 2021 and April 2021 for the post-EDOS implementation group.

Pregnant women and patients with suspected or confirmed COVID-19 infection were excluded. Data were analyzed using two-sample T-test and mixed effects logistic regression. The primary study outcome was the appropriateness of antimicrobials selected, and the secondary outcomes were clinical and microbiologic cure, length of hospital stay, *Clostridioides difficile* infection, and the number of changes in antimicrobial therapy on transition to inpatient setting.

**Results:**

A total of 100 ED patients pre-EDOS implementation and 60 patients post-EDOS implementation were compared. At baseline, patients in the post-EDOS group were older (59.83±20.30 years vs. 50.17±19.97 years, P=0.0037). A higher number of patients in the post-EDOS group had a history of multiple comorbidities (76.67% vs. 54%, P=0.0039). There was a higher rate of appropriate antimicrobial use in the post-EDOS group as compared to the pre-EDOS group (88.3% vs. 50%, P< 0.001). Longer hospital stays were observed in the post-EDOS group (P=0.0005). Clinical cure was similar between the two groups (96.6% vs. 94%, P=0.4568).

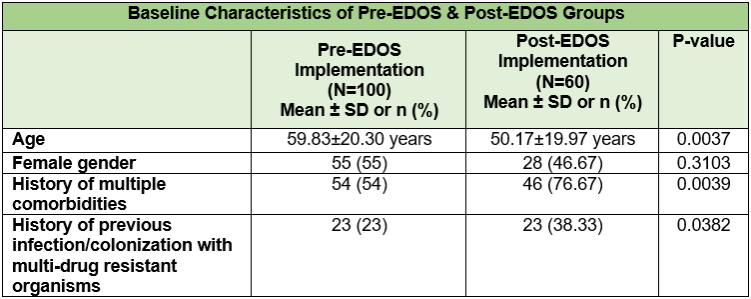

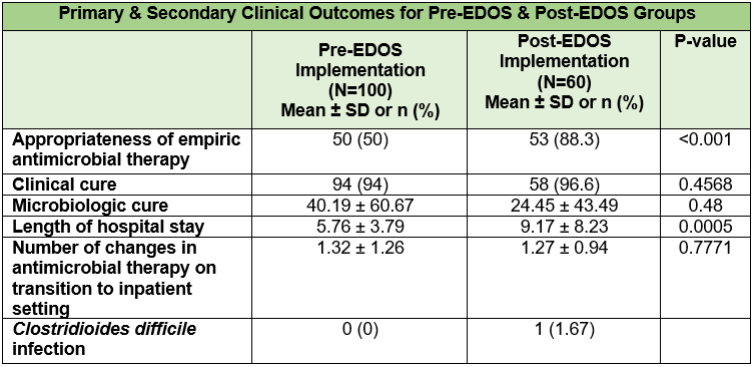

**Conclusion:**

In our study, we observed higher rates of appropriate antimicrobial selection after implementation of an EDOS. Use of an EDOS may represent a valuable AS intervention to guide appropriate antimicrobial prescribing in the ED, and larger studies are needed to confirm those findings.

**Disclosures:**

**All Authors**: No reported disclosures

